# Dihydro-R demonstrates innate immunity against Adenovirus-7 by suppressing the NF-κB/JAK-STAT pathway in a SIRT1-dependent manner

**DOI:** 10.1016/j.bbrep.2025.102387

**Published:** 2025-11-27

**Authors:** Chenyang Wang, Xiaoshan Li, Changbing Wang, Yudan Ye, Mingqi Zhao, Min Guo, Tiantian Xu, Lu Kuang, Yuqing Yan, Wanli Liang, Xingui Tian, Bing Zhu

**Affiliations:** a Central Laboratory, Women and Children's Medical Center Affiliated to Guangzhou Medical University, Guangzhou, Guangdong, China; b State Key Laboratory of Respiratory Disease, National Clinical Research Center for Respiratory Disease, Guangzhou Institute of Respiratory Health, the First Affiliated Hospital of Guangzhou Medical University, Guangzhou Medical University, Guangzhou, 510182, China

**Keywords:** Dihydro-R, Adenovirus, SIRT1, NF-κB pathway, JAK-STAT pathway

## Abstract

Severe adenovirus infections pose significant health challenges, particularly in immunocompromised individuals. This study characterizes the antiviral activity of dihydro-resveratrol (Dihydro-R) against adenovirus type 7 and reveals a SIRT1-dependent mechanism. Our study reveals that Dihydro-R effectively inhibits adenoviral replication across multiple cell lines through SIRT1 activation. Mechanistically, Dihydro-R suppresses the NF-κB and JAK/STAT pathways, leading to reduced expression of inflammatory factors. The critical role of SIRT1 in Dihydro-R's antiviral activity was confirmed through reverse validation using a SIRT1 inhibitor. Notably, Dihydro-R's antiviral effects correlate with SIRT1 upregulation, with A549 cells showing the strongest response. Time-course analysis demonstrates maximal inhibition of NF-κB and JAK/STAT pathways within 48 h of Dihydro-R treatment. Furthermore, Dihydro-R modulates the expression of key cytokines, including IL-8, IL-6, and IL-4, contributing to its anti-inflammatory properties. Our findings not only highlight Dihydro-R as a promising therapeutic candidate for adenovirus infections but also provide insights into SIRT1-targeted antiviral strategies. This study opens new avenues for developing natural compound-based therapies against adenoviral infections and potentially other viral diseases involving similar pathways.

## Highlights

• First demonstration of Dihydro-R's potent antiviral activity against adenovirus type 7 in multiple cell lines (A549, AD293, MRC-5).
• SIRT1-dependent mechanism: Dihydro-R's antiviral effects are mediated through upregulation of SIRT1, confirmed by inhibition with EX-527.
• Dual-pathway modulation: Dihydro-R suppresses adenoviral replication by targeting NF-κB and JAK/STAT signaling pathways, reducing pro-inflammatory cytokines.
• Therapeutic potential: Offers a safer alternative to resveratrol with combined antiviral and anti-inflammatory effects for severe adenovirus infections.

## 1 Introduction

Human adenovirus (HAdV) infections represent a significant global health challenge, occurring year-round regardless of geographical location or season [1]. While these infections are typically self-limiting in immunocompetent individuals, they can cause severe, multi-organ diseases with elevated mortality rates in immunocompromised patients, particularly children [2]. Despite the urgent medical need, there are currently no FDA-approved antiviral drugs specifically targeting HAdV infections [3]. Most existing therapeutic approaches focus on directly targeting viral components, with cidofovir and ribavirin being used off-label as first-line treatments, though their efficacy remains limited and associated with significant side effects [[4], [5], [6], [7], [8], [9]]. Through our systematic screening of traditional Chinese medicine compounds, we identified Dihydro-resveratrol (Dihydro-R) as a promising candidate with potent inhibitory effects against both adenovirus type 7 and type 3 in vitro.

Dihydro-R, a natural polyphenol derived from Polygonum cuspidatum and various fruits, has garnered significant attention due to its diverse therapeutic properties [10,11]. This compound demonstrates remarkable anti-inflammatory effects, improves cardiac function, and provides protection against various pathological conditions [10,12,13]. Unlike conventional antiviral agents that directly target viral components, Dihydro-R exhibits its antiviral activity primarily through modulation of host immune responses. This unique mechanism of action involves the activation of SIRT1, a key regulator of cellular processes including inflammation and antiviral responses [14].

Recent studies have revealed that Dihydro-R's antiviral effects are closely associated with its ability to suppress multiple inflammatory pathways, particularly the NF-κB signaling cascade [15,16]. This mechanism is especially significant as many viruses, including adenoviruses, rely on NF-κB activation for efficient replication [17]. Furthermore, Dihydro-R demonstrates superior bioavailability compared to its precursor resveratrol, potentially offering enhanced therapeutic efficacy [10]. The compound's ability to modulate both inflammatory responses and cellular defense mechanisms through SIRT1-dependent pathways represents a novel approach to antiviral therapy [18]. However, there is still limited direct evidence on the antiviral effect of dihydro-r through SIRT1 activation, which also suggests that this field has great research potential.

Our research aims to elucidate the mechanisms underlying Dihydro-R's antiviral activity against adenovirus, with a particular focus on its interaction with host immune responses through SIRT1-mediated pathways. This study not only advances our understanding of natural compound-based antiviral strategies but also provides valuable insights into the development of broad-spectrum antiviral therapeutics. By investigating Dihydro-R's ability to modulate host immune responses while inhibiting viral replication, we seek to establish a new paradigm in antiviral therapy that could potentially address the current limitations in treating adenovirus infections. The findings from this research could have far-reaching implications for the development of novel therapeutic approaches against various viral infections, particularly in cases where traditional antiviral strategies have proven insufficient. Moreover, our study's focus on host-directed therapy represents a promising alternative to conventional direct-acting antivirals, potentially offering a higher barrier to viral resistance and broader spectrum of activity against multiple adenovirus serotypes.

## 2 Materials and methods

### 2.1 Cells and virus

The adenovirus susceptible cells: A549 cells (Human Lung Adenocarcinoma Cell Line. ATCC# Product code: CRM-CCL-185), AD293 cells (Human Embryonic Kidney (HEK) cell line. ATCC# Product code: CRL-1573) and normal human lung fibroblasts used in this experiment MRC-5 cells (Medical Research Council cell strain 5. ATCC# Product code: CCL-171) were acquired from ATCC. Adenovirus-7 carrying eGFP fluorescence tags were generously provided by Professor Tian Xingui from the National Key Research Institute of Respiratory Diseases at Guangzhou Medical University (Guangzhou, China) [19].

### 2.2 Cell culture and virus infection

Cells were cultivated in a 37 °C cell culture incubator using DMEM medium supplemented with 100U/mL penicillin, 50U/mL streptomycin, and 10 % FBS. Adenovirus-7 was employed for cell infection, and the median tissue culture infectious dose (MOI = 7) was determined using the Reed-Muench method. Upon reaching 80 % cell density, Vero cells were subjected to virus adsorption using adenovirus-7 at a titer of MOI = 7/0.1 mL. Subsequent experiments utilized DMEM supplemented with 1 % fetal bovine serum, 100 U/mL penicillin, and 50 U/mL streptomycin for virus maintenance.

### 2.3 Dihydro-R preparation

Dihydro-R was dissolved in ethanol to create a 100 mM stock solution and stored at −20 °C with glycerol to prevent ice crystal formation. For cell treatments, Dihydro-R was diluted in virus maintenance medium, ensuring the final ethanol concentration remained below 0.5 %.

### 2.4 Cell viability assay

To assess the cytotoxic effects of Dihydro-R, A549, AD293, and MRC-5 cells were cultured with corresponding concentrations of Dihydro-R for 48 h, and cell viability was determined using the CCK-8 assay. The control group consisted of cells incubated with virus maintenance solution (DMEM with 1 % FBS). Cells were seeded at a density of 7 × 10^4^ cells/ml (100 μL) in a 96-well plate, cultured for 12 h, and then treated with various drug concentrations (with ETOH concentration controlled below 0.5 %) for 48 h. Subsequently, an enzyme-linked immunoassay analyzer measured the absorbance at 450 nm after the addition of CCK-8 reagent (10 μL/well) for an additional for 1 h. The specific steps of cell viability testing followed the guidelines in previous articles [20]. For each concentration, three independent biological replicates (n = 3) were used for determination. The absorbance value was used to calculate the percentage of cell viability relative to the control group (set at 100 %). Dose response curves were fitted using logistic regression, and cc50 values were derived from these curves using SPSS 13.0 software. Data are expressed as mean ± standard deviation (SD).

### 2.5 Virus titer determination

MRC-5 cells were cultured in T25 culture flasks using DMEM supplemented with 10 % FBS. When the cell density reached approximately 70–80 %, a clinical isolate virus solution (1 mL) was introduced and placed in a T25 culture flask (430639, Corning, NY, USA). The flask was then placed in a 37 °C, 5 % CO2 incubator, and cytopathic changes were observed daily. Upon achieving cytopathy of 80–90 %, the virus solution was harvested and subjected to consecutive passages. Following two rounds of virus passages, the virus infection titer of the harvested solution was determined on MRC-5 cells using the Reed-Muench two-tier method to calculate the TCID50 value. For this experiment, MRC-5 cells were seeded in a flat-bottomed 96-well cell culture plate at a density of 10^5^ cells/mL and incubated under conditions of 37 °C and 5 % CO2. Then convert to multiplicity of infection (MOI).

### 2.6 Fluorescence microscopy

To confirm that the cytopathic effects (CPE) observed in cells were attributed to HAdV-7 infection, an experiment employed eGFP-adenovirus-7—a variant of HAdV-7 containing the eGFP gene that fluoresces green upon viral replication and proliferation. The MOI = 7 of eGFP-a denovirus-7 was determined on cells using the Reed-Muench two-layer method. Cells were seeded in a flat-bottomed 96-well cell culture plate at a density of 1 × 10^5^ cells/mL and allowed to reach approximately 80 % confluence. Subsequently, eGFP-adenovirus-7, possessing a virulence of MOI = 7, was introduced to the cells and incubated at 37 °C and 5 % CO2 for 2 h. Following incubation, cells were rinsed with PBS to eliminate non-internalized virus particles. At 12, 24, 48, and 72 h post eGFP-adenovirus-7 infection, the CPE status and fluorescence intensity of the cells were assessed through fluorescence microscopy.

### 2.7 Gene enrichment and pathway analysis of Dihydro-R corresponding target proteins

To gain deeper insights into the functional roles of downstream targets of Dihydro-R's target proteins within signaling pathways and gene functions, the list of Dihydro-R target proteins was submitted to the Uniprot database (https://www.uniprot.org). This involved in putting the target gene names and restricting the search to the human species, ensuring that all target gene names were accurately converted to their official gene symbols. Subsequently, the acquired data underwent further analysis with a significance threshold of *P* < 0.05. Enrichment analyses for GO biological processes and KEGG signaling pathways were performed utilizing the METASCAPE platform (https://metascape.org/gp/index.html).

### 2.8 Cytokine Profiles analysis

MRC-5 cells were seeded at a density of 5 × 10^5^/mL in a 10 CM dish. Following a 12-h incubation at 37 °C and 5 % CO_2_, cells were assigned to different groups: the blank control group (virus maintenance solution added), the drug control group (50 μM Dihydro-R), the virus control group (virus concentration of MOI = 7), and the drug and virus treatment group (50 μM Dihydro-R + virus concentration of 100, MOI = 7). After 48 h, supernatant was collected, and cell debris was removed by centrifugation (3000 rpm, 10 min) before analyzing cytokine levels using ELISA. All cytokines were assessed using commercial ELISA kits (BD OptEIATM, Becton Dickinson) following the manufacturer's protocols.

### 2.9 Quantitative RT-PCR

After 48 h of treatment under different conditions, Extract RNA using traditional triazole extraction method and use BeyoFast ™ SYBR Green one-step qRT PCR kit indicates the preparation of reaction solution and RNA amplification reaction. The sequences of the primer pairs were as follows: stat3 forward primer 5′ ACCAGCAGTATAGCCGCTTC 3′ and reverse primer 5′ GCCACAATCCGGGCAATCT 3'. stat1 forward primer 5′ ATCAGGCTCAGTCGGGGAATA 3′ and reverse primer 5′ TGGTCTCGTGTTCTCTGTTCT 3'. stat2 for forward primer 5′ CCAGCTTTACTCGCACAGC 3′ and reverse primer 5’ AGCCTTGGAATCATCACTCCC 3'. Jak1 for forward primer 5′ CTTTGCCCTGTATGACGAGAAC 3′ and reverse primer 5′ ACCTCATCCGGTAGTGGAGC 3'. nf-κb for forward primer 5′ GTTTGTCCAGCTTCGGAGGA 3′ and reverse primer 5′ TGTCACCGCGTAGTCGAAAA 3'. gapdh for rat forward primer 5′ GAAGGTCGGTGTGAACGGAT 3′ and reverse primer 5′ AGTGATGGCATGGACTGTGG 3'.

### 2.10 Western Blot

MRC-5 cells were cultured in a 10 cm dish at a concentration of 5 × 10^5^/mL and subsequently incubated with both HAdV and Dihydro-R for 48 h. Total protein extraction was performed, and protein concentration was quantified using BCA protein kits. The proteins were denatured at 95 °C for 10 min, then separated via 10 % SDS-PAGE gel and transferred onto polyvinylidene difluoride membranes. These membranes were then blocked with 10 % BSA for 2 h and subsequently incubated overnight with primary antibodies, which included SIRT1, p65, p-p65,JAK2, pJAK2,STAT1, pSTAT1,STAT3, pSTAT3, and β-actin antibodies. Following removal of the primary antibodies and subsequent washing, secondary antibodies were applied for a 2 h incubation period. Finally, protein bands were visualized using enhanced chemiluminescent imaging techniques. Refer to our articles for specific methods [21,22].

### 2.11 Co-immunoprecipitation (Co-IP) and immunoblotting

For Co-IP assays, cell lysates were incubated with anti-SIRT1 antibody (or anti-IgG as a negative control) overnight at 4 °C, followed by precipitation with Protein A/G agarose beads. The immunoprecipitates were then washed extensively, eluted, and subjected to Western blot analysis using antibodies against p65, STAT3, and SIRT1.

### 2.12 Statistical analysis

All experiments were conducted with a minimum of three repetitions(n = 3). Results were presented as mean values accompanied by their corresponding standard deviations. Statistical analysis involved variance analysis, and a two-tailed test was performed using SPSS 13.0 software. Significance levels were denoted as follows: *P* < 0.05 (∗), *P* < 0.01 (∗∗), and *P* < 0.001 (∗∗∗), indicating statistically significant differences.

## 3 Results

### 3.1 Cellular toxicity assessment of Dihydro-R

**Fig. 1:**
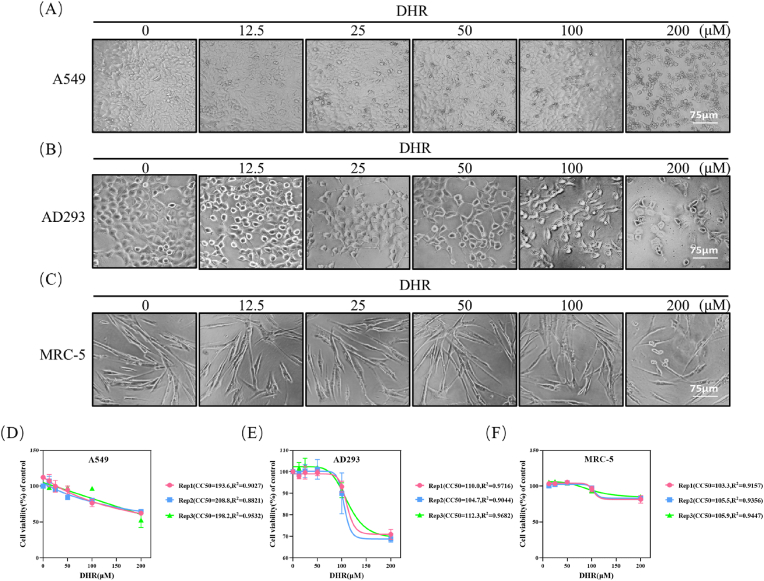
Toxicity of DHR on Diverse Cell Lines. After 48 h of DHR treatment (12.5 μM, 25 μM, 50 μM, 100 μM, 200 μM), the morphology of A549 cells (A), AD293 cells (B), and MRC-5 cells (C) was observed under the microscope. As the drug concentration increases, the cell morphology gradually deviates from normal and begins to become round, wrinkled, or even shed; the scale bar represents 75 μm. The cytotoxic concentration causing 50 % cell death (CC50) was calculated using logistic regression in SPSS 13.0 software. Dose-response curves were plotted, and the relationship between drug concentration and cell viability was evaluated by linear regression. Data points and error bars represent the mean ± SD from three independent experiments (n = 3). CC50 values for A549 (D), AD293 (E), and MRC-5 (F) cells are indicated.

To facilitate our cellular-level antiviral investigations, we conducted toxicity assays using varying concentrations of Dihydro-R (0 μM, 12.5 μM, 25 μM, 50 μM, 100 μM, and 200 μM) on three distinct cell lines: A549, AD293, and MRC-5 (Fig. 1A–C). As the drug concentration increased, we observed a progressive escalation in adverse effects on cells, manifested by reduced mitochondrial dehydrogenase activity leading to diminished reduction of WST-8.

Mitochondrial dehydrogenase activity served as an indicator of cellular viability, and determining Dihydro-R's cytotoxic concentration (CC50) was crucial for establishing a safe and non-toxic concentration range for subsequent experiments. To ensure the reliability of Dihydro-R's cytotoxic concentration (CC_50_) determination, we performed three independent biological replicates for each cell line.Our findings revealed CC50 values of 194.1 μM for A549 cells, 116.0 μM for AD293 cells, and 101.0 μM for MRC-5 cells (Fig. 1D–F). These CC50 values provide critical guidance for ensuring that Dihydro-R is applied within safe and non-toxic parameters in subsequent antiviral studies.

### 3.2 In vitro antiviral activity of Dihydro-R against Adenovirus-7

Following the establishment of Dihydro-R's CC50, we conducted experiments using non-toxic concentration ranges. Cells were infected with MOI = 7 eGFP-adenovirus-7 and simultaneously treated with Dihydro-R. After 48 h, we evaluated the drug's antiviral efficacy by observing fluorescence intensity under a microscope.

**Fig. 2:**
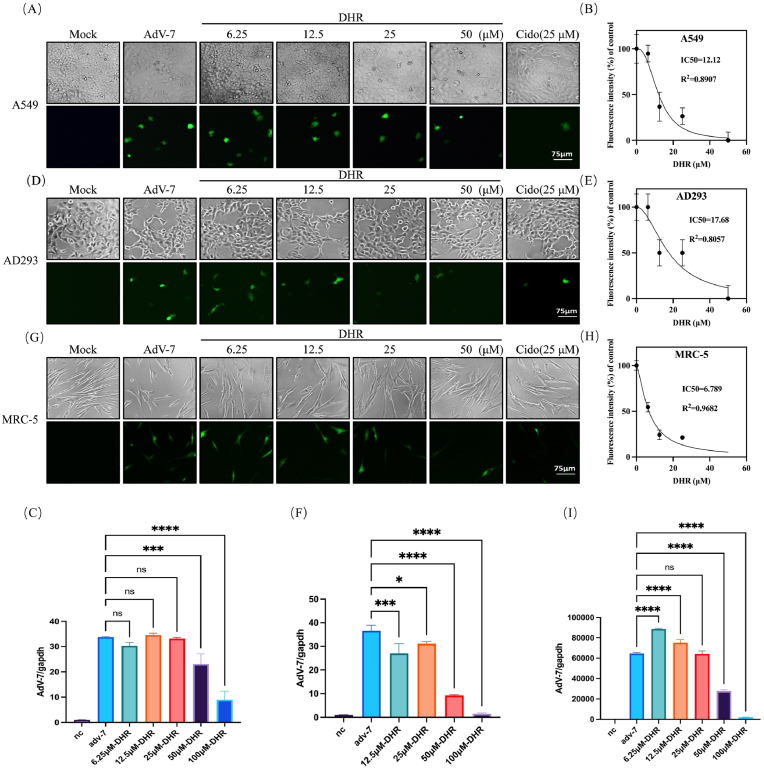
In Vitro Anti-Adenovirus Effect of DHR. DHR gradient dilution for treatment (6.25 μM, 12.5 μM, 25 μM, 50 μM), The cell morphology and fluorescence corresponding to the dark field after 48 h of infection are displayed in A549 cells (A), AD293 cells (D), and MRC-5 cells (G). Due to our observation of virus fluorescence, there is not much variation in cell morphology, the scale bar present 75 μm. The half of the decreased fluorescence point can present DHR's ability to suppress half of the virus (IC50). Use logistic regression to plot dose-response curves and linear regression to evaluate the relationship between drug concentration and cell viability, the IC50 of A549 cells (B); AD293 cells (E); MRC-5 cells (H). n = 3. Quantitative analysis of viral load using PCR of A549 cells (C); AD293 cells (F); MRC-5 cells (I). n = 3.

Dihydro-R demonstrated remarkable inhibitory effects on adenovirus infection across all three cell types, surpassing the positive control-cidofovir at 50 μM. The inhibitory effect intensified with increasing Dihydro-R concentration, as evidenced by the fluorescence spectrum of the virus (Fig. 2A,D, G). Significant inhibition of viruses can also be observed at the genetic level(Fig. 2C,F,I).

Quantification of the inhibitory effect revealed that Dihydro-R suppressed 50 % of the viral concentration (IC50) at 24.94 μM in A549 cells, 39.17 μM in AD293 cells, and 39.46 μM in MRC-5 cells (Fig. 2B,E, H). These findings underscore the potential of Dihydro-R as a potent inhibitor of adenovirus infection across different cell types, with implications for its therapeutic application.

### 3.3 SIRT1 activation: Dihydro-R's modulation of diverse signaling pathways

**Fig. 3:**
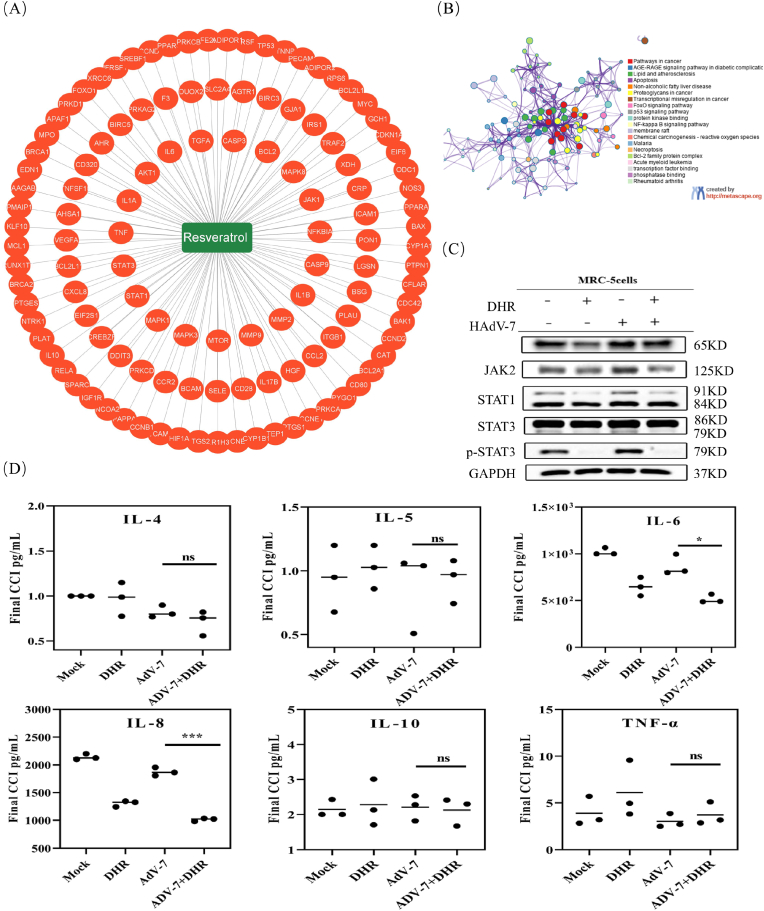
Activation of SIRT1: DHR's Modulation of Multiple Signaling Pathways. The target protein of DHR found on TCMSP website (A) and GO enrichment of these target proteins (B). (C). Extract total protein and analyze the levels of p65, JAK2, STAT1, STAT3, and p-STAT3 proteins through Western Blot. (D). Collect the supernatant and analyze the levels of IL-10, IL-8, IL-6, IL-5, TNF-AIPHA, and IL-4 through ELISA and differential analysis through SPSS.13.0. n = 3. The horizontal lines above the bars specifically denote pairwise comparisons between the virus-infected group (ADV-7) and the ADV-7-infected plus DHR-treated group (ADV-7 + DHR).Significance is indicated by ns, no significance; ∗P ≤ 0.05; ∗∗∗P ≤ 0.001; ∗∗∗∗P ≤ 0.0001.

To elucidate the mechanisms underlying Dihydro-R's antiviral effects, we conducted an exhaustive analysis of proteins associated with Resveratrol, a precursor of Dihydro-R, and the SIRT1 protein, as they share the same active center (Fig. 3A).

GO enrichment analysis revealed a concentration of Resveratrol-related research within cancer, FoxO, NF-κB, and p53 pathways (Fig. 3B). Western Blot experiments demonstrated that Resveratrol reduced protein levels of p65, JAK2, STAT1, STAT3, and phosphorylated STAT3 (Fig. 3C).

Certain viruses depend on NF-κB activation for efficient replication [23]. The inhibition of the NF-κB pathway emerges as a potential mechanism by which Resveratrol enacts its antiviral effects. Resveratrol accomplishes this by obstructing IKK-mediated phosphorylation of IκB, leading to the inhibition of NF-κB nuclear translocation. By regulating the expression of jak and stat, it has a certain effectiveness in exerting antiviral effects. Activation of the JAK2-STAT3 pathway closely associates with inflammation [24].The inhibition of the NF-κB pathway emerges as a potential mechanism by which Resveratrol enacts its antiviral effects. Resveratrol accomplishes this by obstructing IKK-mediated phosphorylation of IκB, leading to the inhibition of NF-κB nuclear translocation.

Our observation noted a decline in IL-10, IL-8, IL-6, IL-5, along with an increase in TNF-α and IL-4 levels in cytokine detection. Statistically significant variations were evident in IL-8, IL-6, and IL-4 (Fig. 3D). These observations contribute to the mediation of the inhibitory impact on NF-κB and JAK-STAT pathways for anti-adenovirus-7 activity.

### 3.4 The antiviral effect of Dihydro-R depends on SIRT1 activation

**Fig. 4:**
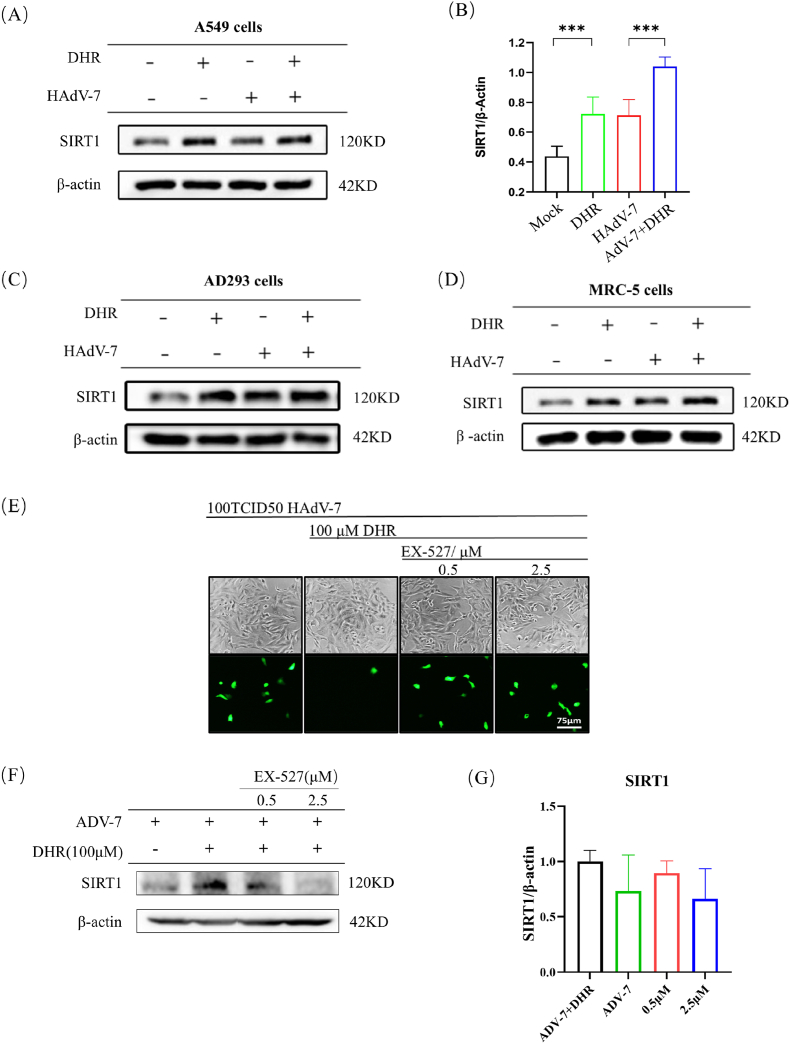
DHR displays antiviral activity through a SIRT1-dependent mechanism. The SIRT1 protein was also detected in MRC-5 cells(A), A549 cells(C) and AD293 cells(D). And quantify the grayscale values(B). (E). The SIRT1 protein inhibitor-EX-527 of was used to observe whether the antiviral effect of DHR was affected by EX-527 under a fluorescence microscope. (F). DHR treated group、DHR treated and added different EX-527 inhibitors group were maintained with virus maintenance solution for 48 h (MOI = 7). Extract total protein and analyze the levels of SIRT1 protein. And quantify the grayscale values(G).

We observed a significant upregulation of SIRT1 protein after Dihydro-R treatment compared to the virus infection group (Fig. 4A and B). This effect was consistent across A549 and AD293 cells (Fig. 4C and D), confirming Dihydro-R's activation effect on SIRT1 with no cell specificity.

To validate the role of SIRT1 in Dihydro-R's antiviral activity [25], for reverse validation, we added the SIRT1 inhibitor-EX-527 to the treatment group to test whether the expression of SIRT1 will influent the antiviral ability of Dihydro-R. The results showed that EX-527 significantly suppressed Dihydro-R's therapeutic effect, leading to viral fluorescence replication (Fig. 4E). EX-527 inhibited SIRT1 protein expression in a concentration-dependent manner (Fig. 4F and G).

### 3.5 The molecular mechanism: DHR-SIRT1 interactions

**Fig. 5:**
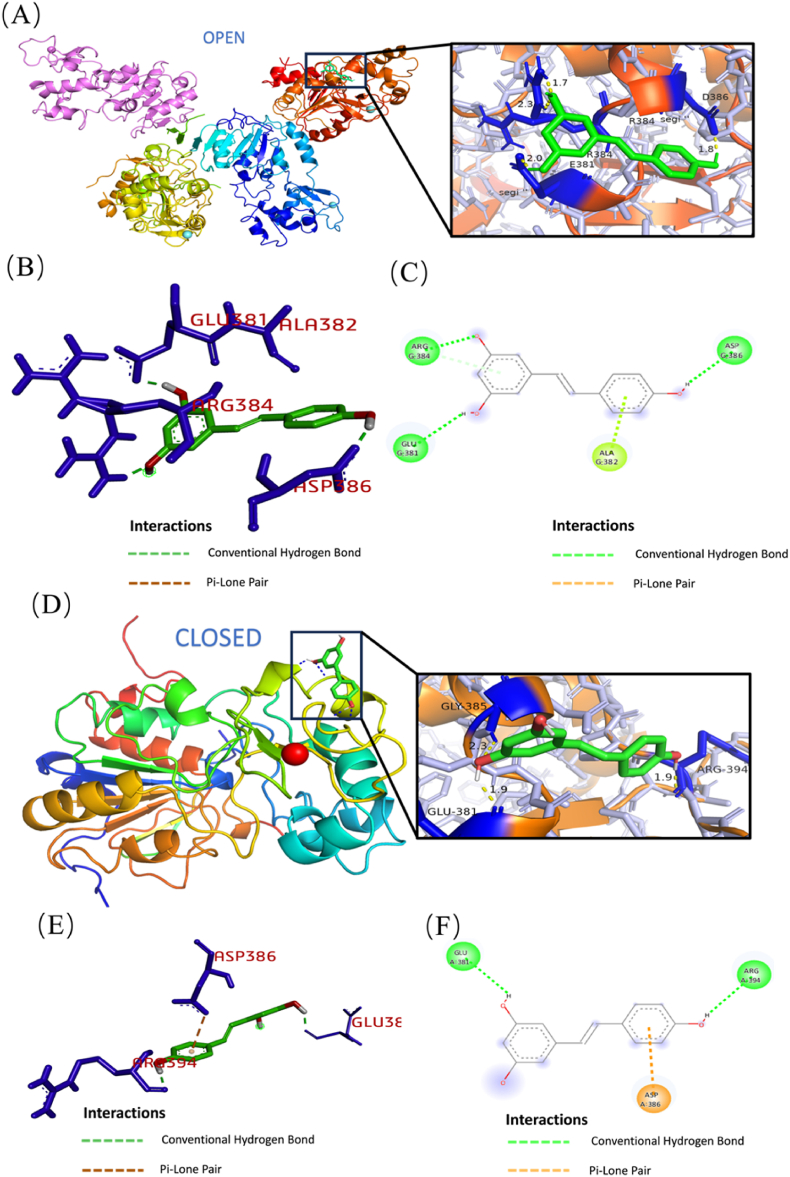
The Molecular Mechanism: Interactions between DHR and SIRT1. Low-energy binding conformations of DHR bound to SIRT1 tetramer in open state protain generated by molecular docking (A), and the 3D (B) and 2D (C) structures of the amino acid sites of its binding part are viewed; to SIRT1 in closed state (D), and the 3D (E) and 2D (F) structures of the amino acid sites of its binding part are viewed.

Our research has revealed a regulatory interaction between DHR, which is known to play a pivotal role in cellular regulation. Building on these findings, we are keen to investigate the molecular dynamics of this interaction further. Specifically, our aim is to elucidate whether DHR directly binds to the SIRT1 protein and to understand the implications of such binding on SIRT1's function. Molecular docking, by simulating the binding process between drug molecules and target proteins, can predict the affinity between the two, i.e., the binding strength. Docking analysis predicts that DHR and SIRT1, in both open and closed states, establish stable binding interactions characterized by reduced binding energy (Fig. 5A,D). The binding energy between DHR and the open-state SIRT1 tetramer is −5.17 kcal/mol, while the binding energy between DHR and the closed-state SIRT1 is −4.52 kcal/mol. Using the FUSTER PRO website [[26], [27], [28], [29], [30]], SIRT1 was docked with its downstream proteins. After introducing the SIRT1 inhibitor (EX-527), a notable increase in viral replication was observed compared to the DHR group. This suggests that the absence of SIRT1 significantly diminishes the antiviral efficacy of DHR (Fig. 5D).

In the open state, DHR is expected to form hydrogen bonds with SIRT1 at the GLU381, ALA382, and ARG384 sites, alongside an additional π-π stacking interaction at the ASP386 site (Fig. 5B and C). Conversely, in the closed state, the GLU381 and ARG394 sites of SIRT1 establish hydrogen bonds with DHR, and an additional π-π stacking interaction occurs at the ASP386 site (Fig. 5E and F). This demonstrates that DHR establishes binding interactions with both the open and closed states of SIRT1. Notably, the open state of SIRT1 contains an additional hydrogen bond compared to the closed state, leading to reduced binding energy.

### 3.6 Dihydro-R therapy downregulates NF-κB/STAT expression at the mRNA and protein levels

**Fig. 6:**
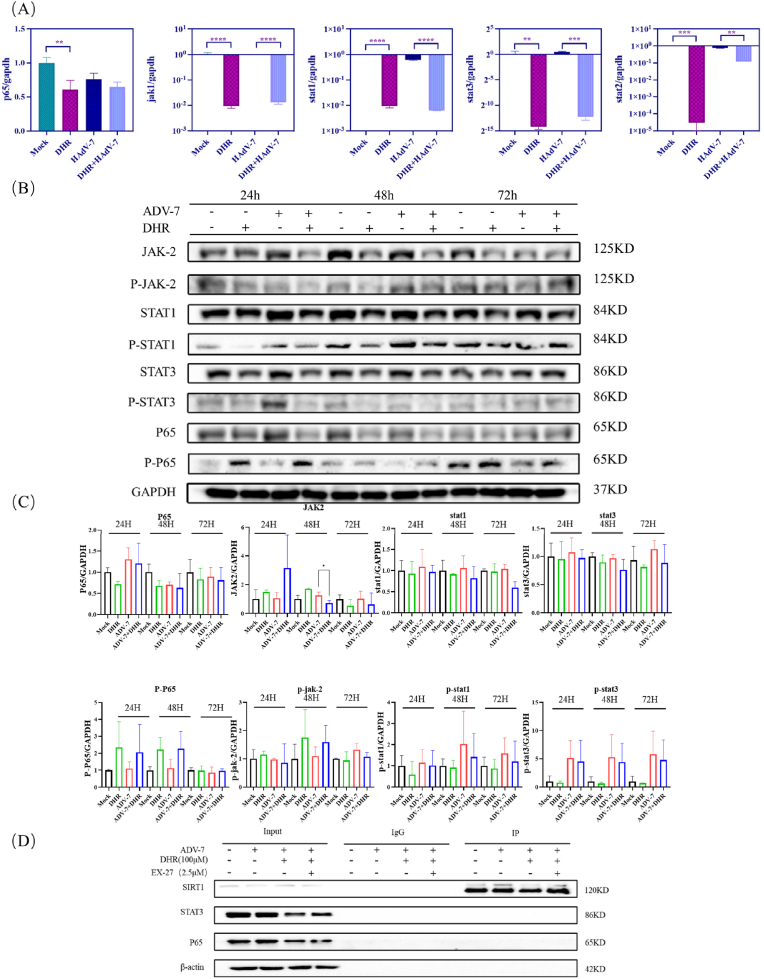
DHR therapy downregulates NF-κB/STAT expression at the mRNA and protein levels(A). Blank control, DHR's control, adenovirus-7 infected control and adenovirus-7 infected and treated with DHR were exposed to virus maintenance solution for 48 h (MOI = 7). Extract total RNA and test the mRNA level of p65, jak1, stat1, stat2, stat3. (B). Blank control, DHR's control, adenovirus-7 infected control and adenovirus-7 infected and treated with DHR were exposed to virus maintenance solution for 48 h (MOI = 7). In three different time periods(24h, 48h, 72h) extract total protein and test the protein level of p65, stat1, stat3, jak2 and their phosphorylation.(C) Semi-quantitative analysis of the protein levels shown in (B). Data are presented as mean ± SD (n = 3). ∗p < 0.05, ∗∗p < 0.01 vs. control group.(D) Co-immunoprecipitation (Co-IP) analysis of the interaction between SIRT1, p65, and STAT3. Cell lysates(48h) were immunoprecipitated with an anti-SIRT1 antibody or control IgG, followed by immunoblotting with the indicated antibodies.

Given the role of NF-κB and JAK/STAT signaling pathways in viral replication and host immune responses [[31], [32], [33]], we investigated whether Dihydro-R modulates these pathways at both transcriptional and protein levels. Quantitative PCR analysis revealed significant downregulation of p65 (a subunit of NF-κB), JAK1, STAT1, STAT2, and STAT3 mRNA levels following Dihydro-R treatment (Fig. 6A). Western blot analysis confirmed that Dihydro-R reduced protein levels of p65, JAK2, STAT1, phosphorylated STAT1,STAT3, and phosphorylated STAT3 in a time-dependent manner (Fig. 6B and C).

The strongest inhibition was observed at 48 h post-treatment, suggesting that Dihydro-R exerts its maximum effect within this timeframe. These findings suggest that the antiviral activity of Dihydro-R involves suppression of NF-κB and JAK/STATsignaling pathways. To further examine whether the functional association between SIRT1 and downstream signaling factors is mediated by direct protein–protein interaction, we conducted co-immunoprecipitation (Co-IP) assays. As shown in Fig. 6D, SIRT1 did not exhibit direct binding to p65 or STAT3, indicating that its regulatory effect is likely exerted through an indirect mechanism.

## 4 Discussions

This study demonstrates the antiviral potential of DHR against adenovirus and identifies its SIRT1-mediated mechanism of action.Our findings reveal a robust inhibitory effect of Dihydro-R on adenoviral replication across multiple cell lines, including A549, AD293, and MRC-5 cells. The antiviral activity of Dihydro-R was found to be dependent on the activation of SIRT1, a key protein involved in various cellular processes. Furthermore, we elucidated the underlying mechanisms by which Dihydro-R exerts its antiviral effects, primarily through the modulation of the NF-κB and JAK/STAT signaling pathways. Adenovirus infection can cause inflammation and upregulation of the JAK/STAT pathway, ultimately leading to increased mRNA levels of IL-6, IL-1 β and IFN - α, as well as increased levels of JAK2, STAT3, *p*-JAK2 and p-STAT3 protein bands [24]. Activation of the JAK2-STAT3 pathway is closely associated with inflammation, and regulation of JAK and STAT expression has been shown to have antiviral effects [34]. Although no stable interaction between SIRT1 and p65 or STAT3 was detected in our Co-IP assays, this finding is consistent with previous studies showing that SIRT1 typically regulates NF-κB and JAK/STAT signalling through indirect mechanisms, such as deacetylation of p65 or p300/CBP and modulation of upstream signalling nodes [[35], [36], [37]].

Our results demonstrate that Dihydro-R significantly upregulates SIRT1 protein levels in all tested cell lines, with A549 cells showing the most pronounced response. This upregulation of SIRT1 correlates strongly with the observed antiviral effects, suggesting a critical role for SIRT1 in mediating Dihydro-R's antiviral activity. The importance of SIRT1 in this process was further confirmed through reverse validation experiments using the SIRT1 inhibitor EX-527, which effectively abolished the antiviral properties of Dihydro-R. These findings align with previous studies that have highlighted the potential of SIRT1 as an antiviral target due to its ability to inhibit inflammatory pathways and regulate antiviral gene expression [38]. Previous literature on the antiviral effects of SIRT1 has only revealed this regulatory mechanism of SIRT1, but has not elucidated why SIRT1 can induce this antiviral effect. In our study, we then investigated the synergistic effects of this regulatory effect and JAK/STAT.

Resveratrol can inhibit the replication of many viruses, including DNA and RNA viruses, and most antiviral studies have focused on the discovery level without much elaboration on the mechanism [[39], [40], [41]]. Some of the studies on antiviral mechanisms have focused on inflammatory pathways, such as the Nrf2/HO-1 pathway associated with sirt1 [42], and others share some similarities with our study, such as the nfkb pathway [43] and inhibition of pro-inflammatory factor expression (IL-6, TNF-α) [44], and inhibition of viral replication through inhibition of the host proteins Protein Kinase B (AKT) and glycogen synthase kinase-3 (GSK-3) [45]. Dihydro-R, a major gut microbiota-derived metabolite of resveratrol, has been reported to exhibit higher bioavailability and multiple biological activities, including anti-inflammatory and anti-tumor effects [46,47]. Our study further reveals its unique antiviral mechanism: Dihydro-R activates SIRT1, which subsequently suppresses NF-κB and JAK/STAT signaling pathways, thereby inhibiting viral replication and modulating inflammatory responses.

**Fig. 7:**
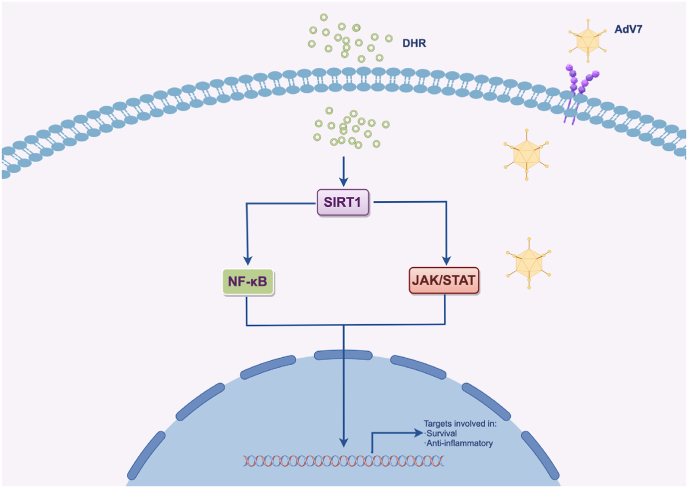
The regulatory role of DHR after entering cells. By Figdraw. ID: IPOUI6df13.

In conclusion, this study demonstrates the potent antiviral activity of Dihydro-R against adenovirus, mediated through SIRT1 activation and subsequent modulation of the NF-κB and JAK/STAT pathways(Fig. 7). These findings have significant implications for both academic research and clinical practice in the field of virology and antiviral therapy. From an academic perspective, our work contributes to the growing body of knowledge on natural compounds with antiviral properties and provides new insights into the molecular mechanisms underlying their effects. In terms of clinical applications, Dihydro-R represents a promising candidate for the development of novel antiviral treatments, particularly for adenovirus infections. Its dual action of inhibiting viral replication while also modulating the inflammatory response could be especially beneficial in managing severe cases of adenovirus infection, where both viral load and excessive inflammation contribute to disease pathology. As we continue to face global health challenges posed by viral pathogens, the development of novel antiviral strategies, such as those based on Dihydro-R, will be crucial in our ongoing efforts to combat infectious diseases and improve public health.

Furthermore, our observation of Dihydro-R's inhibitory effects on the JAK/STAT pathway adds another layer to its antiviral and anti-inflammatory properties. The JAK2-STAT3 pathway is closely associated with inflammation [48], and its suppression by Dihydro-R may contribute to the compound's ability to mitigate the inflammatory response typically associated with viral infections [49]. This dual action of inhibiting viral replication while simultaneously dampening inflammation could make Dihydro-R a particularly promising candidate for treating adenovirus infections, especially in cases where excessive inflammation is a concern.

Despite the promising results, it is important to acknowledge the limitations of our study. Firstly, our investigations were conducted exclusively in vitro, and while they provide valuable insights into the cellular mechanisms of Dihydro-R's antiviral activity, they may not fully capture the complexity of virus-host interactions in vivo. Future studies should aim to validate these findings in animal models of adenovirus infection to better assess the compound's efficacy and safety in a more physiologically relevant context.

Secondly, while we have identified SIRT1 activation as a key mechanism in Dihydro-R's antiviral effects, the full spectrum of cellular pathways and processes influenced by this compound may not have been fully elucidated. Further research using high-throughput screening methods, such as transcriptomics or proteomics, could provide a more comprehensive understanding of Dihydro-R's effects on host cell biology and viral replication. Additionally, the development of a severe adenovirus infection model would be invaluable for assessing the therapeutic potential of Dihydro-R in critically ill patients.

In summary, this study elucidates the antiviral potential of Dihydro-R against adenovirus type 7 and reveals a specific SIRT1-dependent mechanism involving inhibition of the NF-κB and JAK/STAT pathways. While similar signaling axes have been implicated in other viral infections, our findings provide new insight into how a gut microbiota-derived resveratrol metabolite can modulate host innate immunity against adenovirus. These results broaden our understanding of the SIRT1–NF-κB/STAT regulatory network and may inform future antiviral therapeutic strategies.From an academic perspective, our work contributes to the growing body of knowledge on natural compounds with antiviral properties and provides new insights into the molecular mechanisms underlying their effects. In terms of clinical applications, Dihydro-R represents a promising candidate for the development of novel antiviral treatments, particularly for adenovirus infections. Its dual action of inhibiting viral replication while also modulating the inflammatory response could be especially beneficial in managing severe cases of adenovirus infection, where both viral load and excessive inflammation contribute to disease pathology. As we continue to face global health challenges posed by viral pathogens, the development of novel antiviral strategies, such as those based on Dihydro-R, will be crucial in our ongoing efforts to combat infectious diseases and improve public health.

A preprint has previously been published [50].

## Funding

This work was supported by the Basic research project jointly funded by Natural Science Foundation of Guangdong Province, China (2023A1515010589). There was no additional external funding received for this study.

## CRediT authorship contribution statement

**Chenyang Wang:** Data curation, Investigation, Software, Writing – original draft. **Xiaoshan Li:** Investigation, Validation, Writing – review & editing. **Changbing Wang:** Formal analysis, Project administration, Supervision. **Yudan Ye:** Conceptualization, Formal analysis. **Mingqi Zhao:** Conceptualization, Visualization. **Min Guo:** Data curation. **Tiantian Xu:** Investigation. **Lu Kuang:** Validation. **Yuqing Yan:** Software. **Wanli Liang:** Investigation. **Xingui Tian:** Project administration, Resources. **Bing Zhu:** Conceptualization, Funding acquisition, Project administration, Resources.

## Declaration of competing interest

The authors declare that the research was conducted in the absence of any commercial or financial relationships that could be construed as a potential conflict of interest.

## Data Availability

Data will be made available on request.
