# Supplementary material for: Dihydro-R demonstrates innate immunity against Adenovirus-7 by suppressing the NF-κB/JAK-STAT pathway in a SIRT1-dependent manner
Source: Biochem Biophys Rep. 2025 Nov 27;45:102387. doi: 10.1016/j.bbrep.2025.102387 (PMC12702191; doi:10.1016/j.bbrep.2025.102387)
Supplement: Multimedia component 1 [file mmc1.docx]

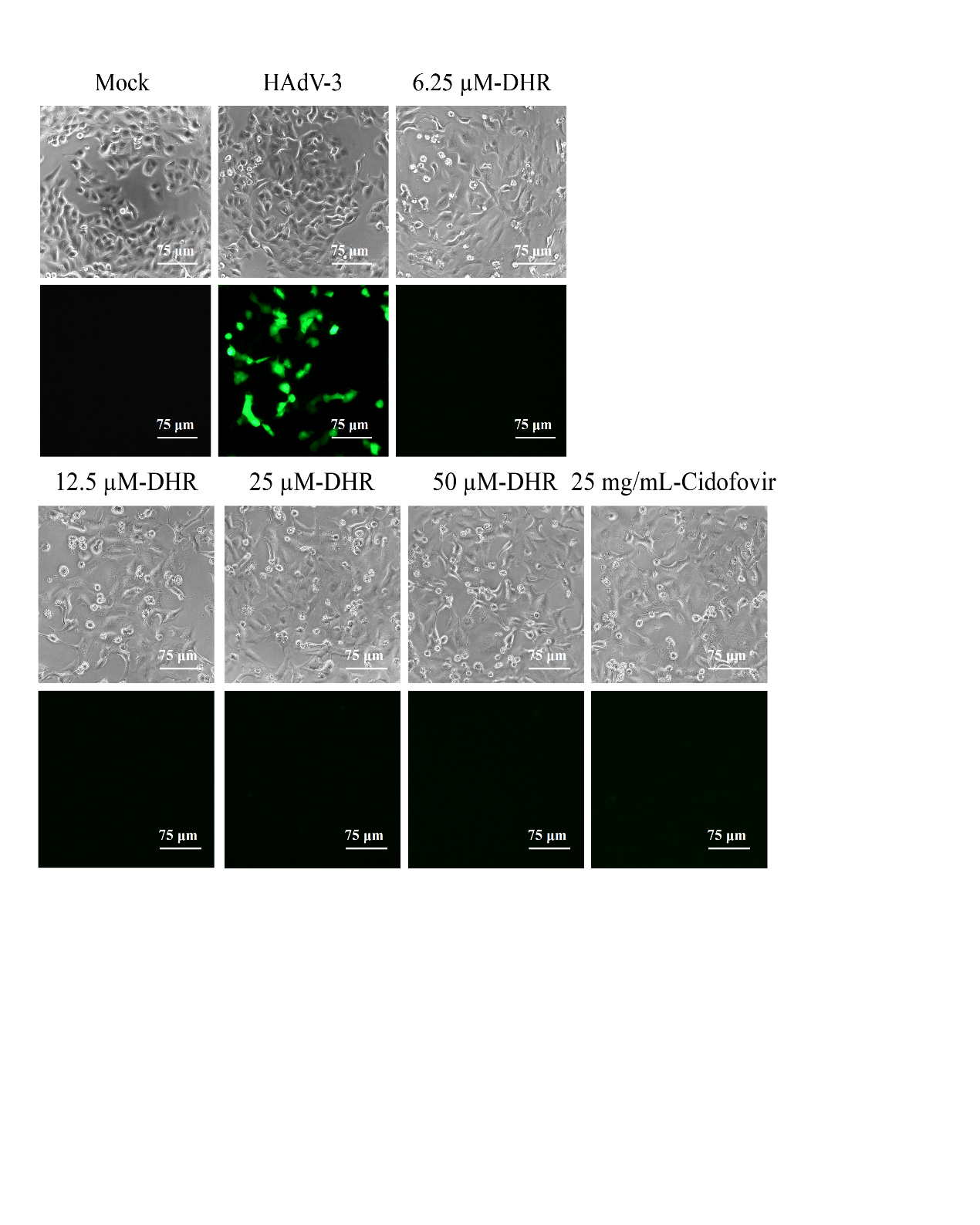


**Figure S1.** Inhibitory effect of Dihydro-R on AdV3 replication in vitro. DHR gradient dilution for treatment (6.25 μM, 12.5 μM, 25 μM, 50 μM), The cell morphology and fluorescence corresponding to the dark field after 48 hours of infection are displayed in A549 cells. Due to our observation of virus fluorescence, there is not much variation in cell morphology, the scale bar present 75µm.
